# Supplementary material for: Cardiovascular risk in subjects over 55 years of age and cognitive performance after five years. NEDICES2-RISK study. Study protocol
Source: PLoS One. 2022 Nov 28;17(11):e0274589. doi: 10.1371/journal.pone.0274589 (PMC9704606; doi:10.1371/journal.pone.0274589)
Supplement: S1 File — (DOCX) [file pone.0274589.s002.docx]

**Título**

Riesgo cardiovascular en mayores de 55 años y rendimiento cognitivo a los 5 años: un modelo de estimación basado en la población española (NEDICES 2)

**Resumen**

**Objetivo principal**. Determinar si existe una asociación significativa entre el riesgo cardiovascular (RCV) y el deterioro cognitivo tras 5 años de seguimiento (de 2014 a 2019) en la población de 55 a 74 años del estudio NEDICES-2 (Neurological Disorders in Central Spain con biobanco).

**Diseño**. Estudio de cohorte observacional analítico longitudinal prospectivo con 5 años de seguimiento

**Ámbito**. Centros de salud de Madrid, Ávila, Salamanca y Segovia

**Sujetos**. Sujetos de 55 a 74 años, adscritos con tarjeta sanitaria a los cupos de los Médicos de Familia del estudio NEDICES-2

**Tamaño de la muestra**. N= 1955 Total observaciones del estudio NEDICES-2

**Determinaciones**. Factores de exposición o predictores (factores de RCV y riesgo calculado con Framingham REGICOR y FRESCO) y variables dependientes (Batería Neuropsicológica Breve, NEDICES-2). Se considerarán como posibles mediadores las variables sociodemográficas, la inteligencia premórbida, el nivel de reserva cognitiva (ocupación, educación y ocio) y el estilo de vida (actividad física y alimentación).

**Estrategia de análisis**. Estudio cualitativo de los datos. Análisis descriptivo de las variables sociodemográficas y clínicas de la muestra. Análisis bivariado de asociación entre el RCV basal y el cambio en los resultados de las pruebas neuropsicológicas (correlación de Pearson y Spearman). Asociación mediante análisis multivariante (ANCOVA) entre RCV basal y cambio en el test BNB-NEDICES, ajustando por diferentes covariables. Estudio y comparación de cambios fiables en muestras independientes.

**Antecedentes**

La llamada transición demográfica es uno de los principales fenómenos sociales de este siglo. El aumento de la población mayor de 80 años conlleva un aumento de las enfermedades crónicas y quienes más atención necesitarán serán los pacientes con enfermedades neurodegenerativas, en particular las demencias. Por ello, la Organización Mundial de la Salud habla no sólo de una transición demográfica, sino también de una transición epidemiológica. Las enfermedades crónicas emergen como la principal causa de muerte en los países desarrollados y son también su problema de salud más prevalente.

A su vez, estas enfermedades están en el origen de la mayor parte de las discapacidades que presentan los individuos y en el aumento de sus necesidades de atención.

La prevalencia de demencia en personas mayores de 60 años está entre el 5-7%. En 2010 había 35,6 millones de personas con demencia y se estima que esta cifra se duplica cada 20 años (1). Pero no solo es una enfermedad común, es una de las mayores causas de invalidez en la vejez y ocupó el puesto número 7 como causa de muerte en 2015 (2).

La demencia es única en términos de su tamaño, costo e impacto. Por lo tanto, incluso un pequeño avance en la prevención de la demencia beneficiaría a millones de personas. Se ha estimado que una intervención que consiguiera retrasar 5 años la aparición de la enfermedad de Alzheimer reduciría la prevalencia en 1,2 millones de personas 10 años después del inicio de la intervención en Estados Unidos (3). Otras estimaciones indican que una reducción del 10 % por década en cada uno de los factores de riesgo daría como resultado una reducción del 8,3 % en la enfermedad de Alzheimer esperada (4).

Varios estudios han informado que la diabetes, la presión arterial alta, la obesidad, el tabaquismo, la depresión, el bajo nivel educativo y la inactividad física son factores de riesgo para la demencia y la enfermedad de Alzheimer, considerando que contribuyen a hasta la mitad de los casos de enfermedad de Alzheimer (5). Esto ha fomentado la investigación en el campo de la prevención de la demencia, como la colaboración de grupos de investigación en la Iniciativa Europea para la Prevención de la Demencia (6). La Organización Mundial de la Salud considera la prevención de la demencia como un elemento clave para contrarrestar la epidemia de demencia (7).

Es de destacar que la mayoría de los estudios sobre factores de riesgo modificables y demencia son observacionales, y los pocos ensayos clínicos existentes muestran resultados dispares. Un ensayo clínico aleatorizado evaluó cómo una intervención multifactorial (dieta, ejercicio físico, entrenamiento cognitivo y control del RCV) mejoró o mantuvo la función cognitiva de personas mayores con alto riesgo cognitivo (8), pero otros ensayos clínicos no lograron demostrar una reducción de la prevalencia de demencia o deterioro cognitivo.

Los datos actuales indican que los factores de RCV aumentan el riesgo de sufrir deterioro cognitivo. No se sabe bien cuándo estos factores de riesgo comienzan a influir en este desempeño (9-13). Diabetes (14), tabaquismo (15) y posiblemente ejercicio (16), e influencia de algunos factores de riesgo en la mediana edad y el posterior desarrollo de deterioro cognitivo y demencia (17), como hipertensión (18), obesidad (19) y la hiperlipidemia (20) son los factores de riesgo más investigados. Otras condiciones necesitan más estudios para hacer las mismas afirmaciones, como la enfermedad coronaria, la insuficiencia renal, la dieta o la actividad cognitiva.

La demencia es también un reto para la salud pública en España. La incidencia de demencia en España es de 12,8 por mil personas y año en pacientes mayores de 65 años (21). Y la prevalencia de factores de riesgo cardiovascular que pueden influir en el rendimiento cognitivo como la hipertensión arterial, la dislipidemia, la obesidad, el tabaquismo y la diabetes mellitus en España es alta (22,23). Pero estudios en población española muestran un riesgo coronario bajo incluso con una alta prevalencia de factores de riesgo cardiovascular en la población (24,25). Esto se ha descrito en la denominada "paradoja mediterránea" o "paradoja francesa" en la que se observan incidencias de enfermedad coronaria inferiores a las esperadas según los factores de riesgo cardiovascular en países del sur de Europa (26). Se han señalado factores genéticos y ambientales como causantes de estas diferencias (26,27). También se sabe que la prevalencia de ictus cerebral en España es inferior a la de otros países (28), y parece que una mayor adherencia a la dieta mediterránea puede disminuir el riesgo de deterioro cognitivo y demencia (29). La relación entre los factores de RCV y el rendimiento cognitivo no ha sido suficientemente investigada en la población española y confirmar su papel como factores de riesgo podría ayudar en la prevención de la demencia.

Las enfermedades crónicas suelen presentar largos períodos de latencia asintomática. Esto es una ventaja para su prevención y tratamiento ya que las intervenciones para retrasar su aparición pueden reducir drásticamente la carga para la sociedad. Identificar qué individuos tienen mayor riesgo de sufrir deterioro cognitivo en los años posteriores mediante el uso de modelos para predecir el RCV puede ser un enfoque útil para desarrollar estrategias para la prevención del deterioro cognitivo. La estimación del RCV a través de diferentes funciones de riesgo, como las de Framingham o el Systematic Coronary Risk Evaluation (SCORE), ha demostrado su utilidad para predecir el rendimiento cognitivo en años posteriores (30-34), encontrando que mayores puntuaciones se relacionaban con un peor rendimiento en test neuropsicológicos. Sin embargo, esto no ha sido suficientemente estudiado en población española. El estudio NEDICES (NEurological DIsorders in Central Spain) (35) comenzó en 1993 y consistió en un estudio longitudinal poblacional que incluyó un total de 5.278 participantes ≥ 65 años de edad, con un seguimiento de 1994 a 2008, y con dos tipos de objetivos: neurológico y general. En 2011 se inició el estudio NEDICES 2 (Neurological Disorders in Central Spain con biobanco)(36), que incluyó sujetos más jóvenes (≥ 55 años) y biobanco asociado (sangre, orina, saliva y cabello). Esta cohorte estaba destinada a evaluar los factores de riesgo y los biomarcadores de las afecciones neurológicas relacionadas con la edad.

Ninguno de los dos estudios NEDICES analizó el riesgo cardiovascular de la población incluida. Por ello, el proyecto para el que se solicita financiación quiere evaluar la población de 55 a 74 años del estudio NEDICES-2 para estudiar la asociación entre el riesgo cardiovascular de los participantes y el deterioro cognitivo en años posteriores. El deterioro cognitivo es la variable de resultado más utilizada en estos estudios, ya que es la etapa previa al deterioro cognitivo.

Además, la relación entre estos factores de riesgo y los indicadores de reserva cognitiva (RC) es relativamente desconocida. Principalmente, la RC consiste en la capacidad de los individuos para optimizar la ejecución a partir de un uso más eficiente de las redes cerebrales. La variabilidad en RC puede deberse a diferencias genéticas y/o eventos experimentados a lo largo de la vida, como educación, cociente intelectual, ocupación o actividades de ocio. Trabajos previos de este equipo de investigación mostraron que ciertos factores relacionados con la RC (educación y ocupación) y la actividad física regular actúan como factores protectores contra el desarrollo de demencia (37,38).

El objetivo del estudio NEDICES2-RISK es investigar la asociación entre el RCV y el cambio en el rendimiento cognitivo tras un seguimiento de 5 años. Para ello se utilizarán las ecuaciones REGICOR (Registre Gironí del Cor) (39) y FRESCO (Función de Riesgo Española de acontecimientos Coronarios y Otros) (40) para la estimación del RCV, basadas en las funciones de Framingham y validadas en población española. junto con la batería neuropsicológica breve desarrollada por el estudio NEDICES2 (41). También se investigará el efecto mediador de la reserva cognitiva y los indicadores de estilo de vida sobre el deterioro cognitivo en diferentes pruebas de la batería neuropsicológica breve del estudio NEDICES-2. Esto permitirá disponer de una cohorte que permita estudiar la influencia del riesgo cardiovascular en el desarrollo del deterioro cognitivo.

Entre las fortalezas de este estudio está el tipo de población que participa en el estudio. Los participantes no serán elegidos en función de sus características de salud (como en otros estudios donde la población participante tiene factores de riesgo cardiovascular u otras características médicas), sino que se incluirán individuos de la población general. Además, la edad elegida de los participantes es mayor de 54 años, mientras que la mayoría de los estudios han evaluado pacientes con una media de edad mayor de 75 años. La inclusión de participantes más jóvenes es fundamental para evaluar cómo el efecto de los factores de riesgo cardiovascular podría influir de manera muy temprana en el inicio del proceso neurodegenerativo que termina en deterioro cognitivo y demencia.

Además, el cálculo del riesgo cardiovascular está ampliamente implantado en España y se realiza de forma rutinaria en las consultas de Atención Primaria. La demostración de que un alto riesgo cardiovascular condicione un peor rendimiento cognitivo en la población española, supondría un avance en el campo de la prevención de las demencias y permitiría definir la población diana óptima para llevar a cabo estrategias de intervención, tal y como ya se hace para la prevención de la enfermedad cardiovascular, sin que ello suponga la aplicación de una nueva herramienta, puesto que ya se utiliza para la prevención de la enfermedad coronaria.

Hay poca conciencia de que el riesgo de demencia puede depender en cierta medida de factores de estilo de vida modificables. La existencia de estudios en población española que avalen la relación entre los factores de riesgo cardiovascular y el deterioro cognitivo podría influir en el abordaje de estos factores de riesgo vascular y en el diseño de estrategias de prevención eficaces. En enfermedades como la demencia que no tienen tratamiento, la prevención es una prioridad.

1. Prince M, Bryce R, Albanese E, Wimo A, Ribeiro W, Ferri CP. The global prevalence of dementia: A systematic review and metaanalysis. Alzheimer’s Dement [Internet]. 2013;9(1):63–75. Available from: http://dx.doi.org/10.1016/j.jalz.2012.11.007

2. World Health Organization. WHO - The top 10 causes of death [Internet]. 24 Maggio. 2018. Available from: https://public.tableau.com/views/who_ghe_mortality_top10_0/TopRankings?:incremente_view_count=no&:embed=y&:loadOrderID=0&:display_count=no&:showTabs=no&:origin=viz_share_link

3. Brookmeyer R, Gray S. Methods for projecting the incidence and prevalence of chronic diseases in ageing populations: application to Alzheimer’s disease. Stat Med [Internet]. 2000 Jun 15;19(11–12):1481–93. Available from: http://www3.interscience.wiley.com/journal/72502511/abstract%5Cnhttp://www.ncbi.nlm.nih.gov/pubmed/10844713

4. Norton S, Matthews FE, Barnes DE, Yaffe K, Brayne C. Potential for primary prevention of Alzheimer’s disease: An analysis of population-based data. Lancet Neurol. 2014;13(8):788–94.

5. Barnes D, Yaffe K. The Projected Impact of Risk Factor Reduction on Alzheimer’s Disease Prevalence. Lancet Neurol. 2013;10(9):819–28.

6. Imtiaz B, Tolppanen A-M, Kivipelto M, Soininen H. Future directions in Alzheimer’s disease from risk factors to prevention. Biochem Pharmacol [Internet]. 2014;88(4):661–70. Available from: http://linkinghub.elsevier.com/retrieve/pii/S0006295214000069

7. WHO. WHO Dementia: a public health priority. World Health Organization 2017. Available from: http://www.who.int/mental_health/neurology/dementia/en/

8. Ngandu T, Lehtisalo J, Solomon A, Levälahti E, Ahtiluoto S, Antikainen R, et al. A 2 year multidomain intervention of diet, exercise, cognitive training, and vascular risk monitoring versus control to prevent cognitive decline in at-risk elderly people (FINGER): a randomised controlled trial. Lancet (London, England) [Internet]. 2015 Jun 6;385(9984):2255–63. Available from: http://www.ncbi.nlm.nih.gov/pubmed/25771249

9. Virta, J. J., Heikkilä, K., Perola, M., Koskenvuo, M., Räihä, I., Rinne, J. O., & Kaprio, J. (2013). Midlife cardiovascular risk factors and late cognitive impairment. *European Journal of Epidemiology*, *28*(5), 405–416. https://doi.org/10.1007/s10654-013-9794-y

10. Plassman, B. L., Jr, J. W. W., Burke, J. R., Holsinger, T., & Benjamin, S. (2009). NIH Conference Annals of Internal Medicine Systematic Review : Factors Associated With Risk for and Possible Prevention of Cognitive Decline in Later Life. *Annals of Internal Medicine*.

11. Whitmer, R. A., Sidney, S., Selby, J., Johnston, S. C., & Yaffe, K. (2005). Midlife cardiovascular risk factors and risk of dementia in late life. *Neurology*, *64*(2), 277–281. https://doi.org/10.1212/01.WNL.0000149519.47454.F2

12. Gorelick, P., Scuteri, a, & Black, S. (2011). contributions to cognitive impairment and dementia a statement for healthcare professionals from the American Heart Association/American Stroke Association. *Stroke*, *42*(9), 2672–2713. https://doi.org/10.1161/STR.0b013e3182299496.Vascular

13. Livingston, G., Sommerlad, A., Orgeta, V., Costafreda, S. G., Huntley, J., Ames, D., … Mukadam, N. (2017). Dementia prevention, intervention, and care. *The Lancet*, *6736*(17). https://doi.org/10.1016/S0140-6736(17)31363-6

14. Cheng G, Huang C, Deng H, Wang H. Diabetes as a risk factor for dementia and mild cognitive impairment: a meta-analysis of longitudinal studies. Intern Med J [Internet]. 2012 May;42(5):484–91. Available from: http://www.ncbi.nlm.nih.gov/pubmed/22372522

15. Anstey KJ, Von Sanden C, Salim A, O’Kearney R. Smoking as a risk factor for dementia and cognitive decline: A meta-analysis of prospective studies. Am J Epidemiol. 2007;166(4):367–78.

16. Barreto P de S, Demougeot L, Vellas B, Rolland Y. Exercise training for preventing dementia, mild cognitive impairment, and clinically meaningful cognitive decline: a systematic review and meta-analysis. Journals Gerontol Ser A [Internet]. 2017;00(00):1–9. Available from: http://academic.oup.com/biomedgerontology/advance-article/doi/10.1093/gerona/glx234/4690262

17. Deckers, K., van Boxtel, M. P. J., Schiepers, O. J. G., de Vugt, M., Muñoz Sánchez, J. L., Anstey, K. J., … Köhler, S. (2015). Target risk factors for dementia prevention: a systematic review and Delphi consensus study on the evidence from observational studies. *International Journal of Geriatric Psychiatry*, *30*(3), 234–246. https://doi.org/10.1002/gps.4245

18. Iadecola C, Yaffe K, Biller J, Bratzke LC, Faraci FM, Gorelick PB, et al. Impact of Hypertension on Cognitive Function: A Scientific Statement From the American Heart Association. [Internet]. Vol. 68, Hypertension (Dallas, Tex. : 1979). 2016. 67–94 p. Available from: http://www.ncbi.nlm.nih.gov/pubmed/27977393%0Ahttp://www.pubmedcentral.nih.gov/articlerender.fcgi?artid=PMC5361411

19. Albanese E, Launer LJ, Egger M, Prince MJ, Giannakopoulos P, Wolters FJ, et al. Body mass index in midlife and dementia: Systematic review and meta-regression analysis of 589,649 men and women followed in longitudinal studies. Alzheimer’s Dement (Amsterdam, Netherlands) [Internet]. 2017;8:165–78. Available from: http://linkinghub.elsevier.com/retrieve/pii/S2352872917300374

20. Anstey KJ, Lipnicki DM, Low L-F. Cholesterol as a risk factor for dementia and cognitive decline: a systematic review of prospective studies with meta-analysis. Am J Geriatr Psychiatry [Internet]. 2008;16(5):343–54. Available from: http://www.ncbi.nlm.nih.gov/pubmed/18448847

21. Bermejo-Pareja, F., Llamas-Velasco, S., & Villarejo-Galende, A. (2016). Alzheimer’s disease prevention: A way forward. *Revista Clinica Espanola*, *216*(9), 495–503. https://doi.org/10.1016/j.rce.2016.05.010

22. Grau M, Elosua R, Cabrera de León A, Guembe MJ, Baena-Díez JM, Vega Alonso T, et al. [Cardiovascular risk factors in Spain in the first decade of the 21st Century, a pooled analysis with individual data from 11 population-based studies: the DARIOS study]. Rev Esp Cardiol [Internet]. 2011 Apr;64(4):295–304. Available from: https://linkinghub.elsevier.com/retrieve/pii/S0300893211001515

23. Medrano, M. J., Cerrato, E., Boix, R., & Delgado-Rodríguez, M. (2005). Factores de riesgo cardiovascular en la población española: metaanálisis de estudios transversales. *Medicina clínica*, *124*(16), 606-612.

24. Marín A, Medrano MJ, González J, Pintado H, Compaired V, Bárcena M, et al. Risk of ischaemic heart disease and acute myocardial infarction in a Spanish population: Observational prospective study in a primary-care setting. BMC Public Health. 2006;6:1–11.

25. Masiá R, Pena A, Marrugat J, Sala J, Vila J, Pavesi M, et al. High prevalence of cardiovascular risk factors in Gerona, Spain, a province with low myocardial infarction incidence. REGICOR Investigators. J Epidemiol Community Health [Internet]. 1998;52(11):707–15. Available from: http://www.pubmedcentral.nih.gov/articlerender.fcgi?artid=1756647&tool=pmcentrez&rendertype=abstract

26. Ferrieres J. The French paradox: lessons for other countries. Heart [Internet]. 2004;90(1):107–11. Available from: http://heart.bmj.com/cgi/doi/10.1136/heart.90.1.107

27. Lao O, Dupanloup I, Barbujani G, Bertranpetit J, Calafell F. The mediterranean paradox for susceptibility factors in coronary heart disease extends to genetics. Ann Hum Genet. 2008;72(1):48–56.

28. Vega T, Zurriaga O, Ramos JM, Gil M, Álamo R, Lozano JE, et al. Stroke in Spain: Epidemiologic Incidence and Patterns; A Health Sentinel Network Study. J Stroke Cerebrovasc Dis. 2009;18(1):11–6.

29. Lourida I, Soni M, Thompson-Coon J, Purandare N, Lang IA, Ukoumunne OC, et al. Mediterranean diet, cognitive function, and dementia: A systematic review. Epidemiology. 2013;24(4):479–89.

30. Harrison SL, Ding J, Tang EYH, Siervo M, Robinson L, Jagger C, et al. Cardiovascular disease risk models and longitudinal changes in cognition: A systematic review. PLoS One. 2014;9(12):1–14.

31. Dregan A, Stewart R, Gulliford MC. Cardiovascular risk factors and cognitive decline in adults aged 50 and over: a population-based cohort study. Age Ageing [Internet]. 2013 May;42(3):338–45. Available from: http://www.ncbi.nlm.nih.gov/pubmed/23179255

32. Kaffashian S, Dugravot A, Nabi H, Batty GD, Brunner E, Kivimki M, et al. Predictive utility of the Framingham general cardiovascular disease risk profile for cognitive function: Evidence from the Whitehall II study. Eur Heart J. 2011;32(18):2326–32.

33. Viticchi G, Falsetti L, Buratti L, Boria C, Luzzi S, Bartolini M, et al. Framingham risk score can predict cognitive decline progression in Alzheimer’s disease. Neurobiol Aging. 2015;36(11):2940–5.

34 DeRight J, Jorgensen RS, Cabral MJ. Composite Cardiovascular Risk Scores and Neuropsychological Functioning: A Meta-Analytic Review. Ann Behav Med. 2015;49(3):344–57.

35. Bermejo-Pareja F, Benito-León J, Vega-Q S, Díaz-Guzmán J, Rivera-Navarro J, Molina JA, et al. [The NEDICES cohort of the elderly. Methodology and main neurological findings]. Rev Neurol [Internet]. 2008;46(7):416–23. Available from: http://www.ncbi.nlm.nih.gov/pubmed/18389461

36. Hernández-Gallego J, Llamas-Velasco S, Bermejo-Pareja F, Vega S, Tapias-Merino E, Rodríguez-Sánchez E, et al. Neurological Disorders in Central Spain, Second Survey: Feasibility Pilot Observational Study. JMIR Res Protoc 2019;8(1)e10941 https//www.researchprotocols.org/2019/1/e10941/ [Internet]. 2019 Jan 10 [cited 2019 Feb 8];8(1):e10941. Available from: https://www.researchprotocols.org/2019/1/e10941/

37. Llamas-Velasco S, Contador I, Villarejo-Galende A, Lora-Pablos D, Bermejo-Pareja F. Physical Activity as Protective Factor against Dementia: A Prospective Population-Based Study (NEDICES). J Int Neuropsychol Soc [Internet]. 2015 Nov 19 [cited 2018 Feb 14];21(10):861–7. Available from: http://www.journals.cambridge.org/abstract_S1355617715000831

38. Contador I, Bermejo-Pareja F, Puertas-Martin V, Benito-Leon J. Childhood and Adulthood Rural Residence Increases the Risk of Dementia: NEDICES Study. Curr Alzheimer Res [Internet]. 2015 Apr 27;12(4):350–7. Available from: http://www.eurekaselect.com/openurl/content.php?genre=article&issn=1567-2050&volume=12&issue=4&spage=350

39. Marrugat J, Vila J, Baena-Díez JM, Grau M, Sala J, Ramos R, et al. [Relative validity of the 10-year cardiovascular risk estimate in a population cohort of the REGICOR study]. Rev Esp Cardiol [Internet]. 2011;64(5):385–94. Available from: http://www.revespcardiol.org/es/validez-relativa-estimacion-del-riesgo/articulo/90003647/

40. Marrugat, J., Subirana, I., Ramos, R., Vila, J., Marín-Ibañez, A., Guembe, M. J., … Elosua, R. (2014). Derivation and validation of a set of 10-year cardiovascular risk predictive functions in Spain: The FRESCO Study. *Preventive Medicine*, *61*, 66–74. https://doi.org/10.1016/j.ypmed.2013.12.031

41. Serna, A., Contador, I., Bermejo-Pareja, F., Mitchell, AJ, Fernandez-Calvo, B., Ramos, F., ... & Benito-Leon, J. (2015). Precisión de una batería neuropsicológica breve para el diagnóstico de demencia y deterioro cognitivo leve: un análisis de la cohorte NEDICES. *Diario de la enfermedad de Alzheimer* , *48* (1), 163-173.

**Hipótesis**

Un mayor riesgo cardiovascular en la población de 55 a 74 años, medido con las ecuaciones de riesgo de Framingham REGICOR y FRESCO, se relaciona con una peor puntuación a los 5 años de seguimiento en la Batería Neuropsicológica Breve utilizada en el estudio NEDICES2.

**Objetivos**

**Objetivo principal**

Determinar la relación entre el RCV, medido con las ecuaciones de riesgo de Framingham REGICOR y FRESCO, y el cambio en el rendimiento cognitivo a los 5 años en los sujetos de 55 a 74 años incluidos en el estudio NEDICES2-RISK.

**Objetivos secundarios**

a) Describir el perfil de los pacientes en función de su RCV.

(b) Evaluar la asociación entre cada uno de los factores de RCV estudiados y el cambio en el rendimiento cognitivo a los 5 años.

(c) Analizar los posibles factores mediadores entre el RCV, el rendimiento cognitivo y el estilo de vida.

(d) Evaluar el efecto de los indicadores de RC (inteligencia verbal, educación, ocupación y estilo de vida) sobre el rendimiento cognitivo, medido a través de diferentes pruebas neuropsicológicas.

**Métodos/diseño**

**Diseño**. Estudio de cohorte prospectivo, analítico, observacional con un seguimiento de 5 años.

**Ámbito**. El reclutamiento de pacientes se llevó a cabo en el ámbito de atención primaria dentro del Sistema Nacional de Salud español en las regiones de Ávila, Madrid, Salamanca y Segovia, España.

**Población**. La población estudiada son pacientes de 55 a 74 años, que fueron incluidos en la cohorte del estudio anterior NEDICES2 (2014-2017). La población del estudio NEDICES2 se originó a partir de la lista de usuarios (titulares de la tarjeta de la seguridad social) de los médicos participantes en los centros de salud incluidos, quienes luego fueron seleccionados mediante un muestreo aleatorio de pacientes ≥ 55 años estratificados por sexo y edad de 5 en 5 años.

**Criterios de selección**

1. Criterios de inclusión

• Edad de 55 a 74 años

• Proporcionar consentimiento informado por escrito para su inclusión en el estudio

2. Criterios de exclusión

• Presentar un diagnóstico de demencia al inicio

**Tamaño de la muestra**: 1955. Todos los sujetos del estudio NEDICES2.

**Variables**

a) Variables sociodemográficas: edad, sexo, nivel educativo, ocupación actual, estado civil, peso al nacer, unidad familiar y antecedentes familiares. Hábitos de salud: horas de sueño, consumo de alcohol, consumo de tabaco, actividad física y dieta mediterránea. Cuestionario de depresión: CES-D. Enfermedades crónicas actuales y tratamientos recogidos y confirmados por médicos de familia. Test de acentuación de palabras.

b) Variables de exposición: Factores de riesgo cardiovascular. Riesgo cardiovascular medido con las ecuaciones de riesgo de Framingham REGICOR y FRESCO en el momento de la primera evaluación neuropsicológica realizada en NEDICES2 (2014) y 5 años después.

c) Principales variables de resultado: versión de 37 ítems del Mini-Mental State Examination (MMSE-37), memoria inmediata y diferida medida a través de las ilustraciones SEN-FIS, versión de 11 ítems del Functional Activities Questionnaire (FAQ) de Pfeffer, índice de Katz de Independencia en las Actividades de la Vida Diaria (AVD), Trail Making Test, prueba de fluidez oral por categorías (categoría: animales) y test del reloj.

**Recopilación de datos**

La recogida de datos se realiza en dos momentos diferentes:

1. Variables registradas en el estudio NEDICES2 hace 3 años (2014):

a. Entrevistas y cuestionarios realizados por el médico de familia en el estudio NEDICES2: enfermedades crónicas y medicación prescrita en el último mes

b. Entrevistadores entrenados: variables sociodemográficas y pruebas neuropsicológicas

C. Variable de exposición: el cálculo del riesgo cardiovascular se realizará con los datos anteriores

2. Variables recogidas en 2019:

a. Factores de riesgo cardiovascular recogidos por los médicos de familia en 2019

b. Entrevistadores entrenados: variables sociodemográficas y pruebas neuropsicológicas

C. Variable de exposición: nuevo cálculo del riesgo cardiovascular en 2019

**Análisis estadístico**

1. Se comprobará la calidad de los datos: errores de codificación, missing data y posibles sesgos relacionados con la representatividad de la muestra.

2. Análisis descriptivo. Variables sociodemográficas y clínicas de los sujetos: frecuencias y porcentajes en el caso de variables categóricas, y por sus medias (desviación estándar) o mediana (rango intercuartílico) en el caso de variables cuantitativas.

3. Análisis bivariado: relación entre RCV basal (cuatro categorías: riesgo cardiovascular bajo, medio, alto y muy alto) y la puntuación obtenida en las pruebas neuropsicológicas, MMSE-37, memoria inmediata y diferida medida a través de las ilustraciones SEN-FIS, versión de 11 ítems del Cuestionario de Actividades Funcionales (FAQ) de Pfeffer, Trail Making Test, prueba de fluidez oral por categorías (categoría: animales) y prueba de dibujo del reloj.

4. Análisis multivariante: relación entre RCV basal y cambio en pruebas neuropsicológicas ajustadas por variables sociodemográficas y clínicas relevantes y posibles factores de confusión mediante ANCOVA. Los modelos de efectos mixtos multinivel de regresión lineal se ajustarán para tener en cuenta la variabilidad intra-sujeto (tomando medidas repetidas) y la agrupación de pacientes.

5. Se analizará el cambio fiable para cada prueba en muestras independientes (riesgo cardiovascular alto vs riesgo cardiovascular bajo) utilizando el método de Hsu y Chelene. Se presentarán tablas de cambio normalizadas para cada uno de los grupos controlando el efecto de las variables sociodemográficas.

**Limitaciones**

En los casos de falta de datos se utilizará la información del expediente clínico de los pacientes. Utilizar los datos de una fuente secundaria de información puede afectar la calidad de los datos de variables como la presión arterial, el peso, la altura o el estilo de vida, ya que pueden faltar o por la variabilidad resultante de la recopilación por múltiples profesionales. Sin embargo, trabajar con expedientes clínicos electrónicos, sistema implantado desde hace más de 15 años, garantiza la calidad y homogeneidad de los datos que contienen.

Los factores de RCV pueden cambiar a lo largo del periodo estudiado, por no estar más expuestos a ellos o por la aparición de otros nuevos. Para evitar el error que estos cambios pueden producir, se estimará el RCV en dos momentos, en la primera evaluación neuropsicológica y cinco años después en la segunda, y se examinarán las posibles variaciones entre ellos.

**Consideraciones éticas y confidencialidad de los datos**

Se cuenta con el consentimiento informado de los pacientes para el estudio NEDICES2. Se solicitará permiso para revisar la documentación e historia clínica de los participantes al Comité de Ética en Investigación del Hospital 12 de Octubre.

Existe una página web dirigida a los pacientes con el objetivo de facilitarles información relacionada con el estudio, así como facilitarles los principales hallazgos y resultados del estudio.

Las estimaciones de RCV y MMSE-37 serán entregadas a los médicos de atención primaria responsables de los pacientes incluidos para ajustar su tratamiento si es necesario o profundizar en su evaluación del rendimiento cognitivo.

Se creará una base de datos local para registrar todos los datos obtenidos. Al finalizar este estudio, esta base de datos se enviará a la base de datos central del estudio NEDICES2, y una copia se mantendrá en el Departamento de Neurología del Hospital 12 de Octubre. La base de datos nominativa estará protegida por contraseñas solo disponibles para los investigadores.

Se garantizará la confidencialidad y el anonimato de los datos según la Ley 15/1999 de confidencialidad de datos, tanto en la fase de ejecución del proyecto como en las presentaciones o publicaciones derivadas del mismo.
