# Supplementary material for: Cardiovascular risk in subjects over 55 years of age and cognitive performance after five years. NEDICES2-RISK study. Study protocol
Source: PLoS One. 2022 Nov 28;17(11):e0274589. doi: 10.1371/journal.pone.0274589 (PMC9704606; doi:10.1371/journal.pone.0274589)
Supplement: S2 File — (DOCX) [file pone.0274589.s003.docx]

**Title**

Cardiovascular risk in people older than 55 years and cognitive performance at 5 years: an estimation model based on the Spanish population (NEDICES 2)

**Abstract**

**Main objective**. To determine if there is a significant association between cardiovascular risk (CVR) and cognitive decline after 5 years of follow-up (from 2014 to 2019) in the population aged 55 to 74 years of the NEDICES-2 study (Neurological Disorders in Central Spain with bio-bank).

**Design**. Prospective longitudinal analytical observational cohort study with 5 years of follow-up

**Area**. Health centers from Madrid, Avila, Salamanca y Segovia

**Subjects.** Subjects from 55 to 74 years of age, assigned with a health card to the quotas of Family doctors in the NEDICES-2 study

**Sample size.** N= 1955 Total observations of the NEDICES-2 study

**Determinations.** Exposure factors or predictors (factors of CVR and risk calculated with Framingham REGICOR and FRESCO) and dependent variables (Brief Neuropychological Battery, NEDICES-2). The sociodemographic variables, premorbid intelligence, the leve lof cognitive reserve (occupation, education and leisure) and lifestyle (physical activity and diet) will be considered as possible mediators

**Analysis strategy.** Study of data qualitly.Descriptive Analysis soviodemographic and clinical variables of the sample. Bivariate analysis associatioin between baseline CVR and change in neuropsychological test results (Pearson and Spearman correlation). Multivariate analysis (ANCOVA) association between baseline CVR and change in the BNB-NEDICES test, adjusting for different covariates. Study and comparison of reliable change in independent samples.

**Background**

The so-called demographic transition is one of the main social phenomena of this century. The increase in the population over 80 years of age leads to an increase in chronic diseases and those who will need the most attention will be patients with neurodegenerative diseases, particularly dementia. For this reason, the World Health Organization speaks not only of a demographic transition, but also of an epidemiological transition. Chronic diseases emerge as the main cause of death in developed countries and are also their most prevalent health problem; In turn, these diseases are at the origin of most of the disabilities that individuals present and in the increase in their care needs.

The prevalence of dementia in people over 60 years of age is between 5-7%. In 2010 there were 35.6 million people with dementia and it is estimated that this figure doubles every 20 years (1). But not only is it a common disease, it is one of the greatest causes of disability in old age and ranked number 7 as a cause of death in 2015 (2).

Dementia is unique in terms of its size, cost and impact. Therefore, even a small advance in dementia prevention would benefit millions of people. It has been estimated that an intervention that succeeded in delaying the onset of Alzheimer's disease by 5 years would reduce the prevalence by 1.2 million people 10 years after the start of the intervention in the United States (3). Other estimates state that a 10% reduction per decade in each of the risk factors would result in an 8.3% reduction in expected Alzheimer's disease (4).

Several studies have reported diabetes, high blood pressure, obesity, tobacco addiction, depression, low educational level, and physical inactivity as risk factors for dementia and Alzheimer’s disease, whereas they contribute to up to half of Alzheimer's disease cases (5). This has promoted research in the field of preventing dementia, such as the collaboration of research groups in the European Dementia Prevention Initiative (6). The World Health Organization considers the prevention of dementia as a key element to counteract the dementia epidemic (7).

Of note, most studies on modifiable risk factors and dementia are observational, and the few existing clinical trials show disparate results. A randomized clinical trial reported that a multi-factor intervention (diet, physical exercising, cognitive training, and CVR control) improved or maintained the cognitive function of the elderly with high cognitive risk (8), but other clinical trials have failed to demonstrate a reduction in the prevalence of dementia or cognitive deterioration.

Current data indicate that CVR factors increase the risk of suffering cognitive deterioration. It is not well known when these risk factors begin to influence this performance (9-13). Diabetes (14), smoking (15), and possibly exercise (16), and influence of some risk factors in middle age and the subsequent development of cognitive impairment and dementia (17), such as hypertension (18), obesity (19) and hyperlipidemia (20) are the most investigated risk factors. Other conditions need more studies to make the same claims, such as coronary heart disease, kidney failure, diet, or cognitive activity.

Dementia is also a challenge for public health in Spain. The incidence of dementia in Spain is 12.8 per thousand people per year in patients over 65 years of age (21). And the prevalence of cardiovascular risk factors that can influence cognitive performance such as high blood pressure, dyslipidemia, obesity, smoking and diabetes mellitus in Spain is high (22,23). But studies in the Spanish population show a low coronary risk even with a high prevalence of cardiovascular risk factors in the population (24,25). This has been described in the so-called "Mediterranean paradox" or "French paradox" in which incidences of coronary heart disease are observed to be lower than expected according to cardiovascular risk factors in southern European countries (26). Genetic and environmental factors have been pointed out as causing these differences (26,27). The prevalence of brain stroke in Spain is also known to be lower than that in other countries (28), and it appears that greater adherence to the Mediterranean diet can decrease the risk of cognitive deterioration and dementia (29). The relationship between CVR factors and cognitive performance has not been sufficiently investigated in the Spanish population and confirming their role as risk factors could help in the prevention of dementia.

Chronic conditions usually present long, asymptomatic latency periods. This is an advantage for their prevention and treatment since interventions for delaying their onset can drastically reduce the burden for society. Identifying which individuals are at higher risk of suffering cognitive deterioration in subsequent years by using models for predicting CVR can be a useful approach for developing strategies for the prevention of cognitive impairment. Estimating CVR through different risk functions, such as the Framingham’s risk score or the Systematic Coronary Risk Evaluation (SCORE), has proven its usefulness for predicting cognitive performance in subsequent years (30–34), finding that greater scores were related poorer performance in neuropsychological tests. However, this has not been sufficiently studied in the Spanish population.he study NEurological DIsorders in CEntral Spain (NEDICES) (35) commenced in 1993 and consisted of a population, longitudinal study including a total of 5,278 participants ≥65 years of age, with a follow-up from 1994 to 2008, and with two types of objectives: neurologic and general. In 2011, the study NEurological DIsorders in CEntral Spain with biobank (NEDICES2) (36) began, which included younger subjects (≥55 years) and incorporated the services of a biobank (blood, urine, saliva, and hair). This cohort was meant to assess risk factors and biomarkers of age-related neurologic conditions.

Neither of the two NEDICES studies analyzed the cardiovascular risk of the included population. For this reason, the project for which funding is requested wants to assess the population aged 55 to 74 years of the NEDICE-2 study to study the association between the cardiovascular risk of the participants and cognitive decline in later years. Cognitive decline is the most used outcome variable in these studies, since it is the stage prior to cognitive decline.

Additionally, the relationship between these risk factors and indicators of cognitive reserve (CR) is relatively unknown. Mainly, the CR consists of the ability of individuals to optimize execution based on a more efficient use of brain networks. The variability in CR can be due to genetic differences and/or events experienced throughout life such as education, intelligence quotient, occupation, or leisure activities. Previous work by this research team showed that certain factors related to CR (education and occupation) and regular physical activity act as protective factors against developing dementia (37,38).

The aim of the study NEDICES2-RISK is to investigate the association between CVR and the change in cognitive performance after a 5-year follow-up. For this, the REGICOR (Registre Gironí del Cor) (39) and FRESCO (Función de Riesgo Española de acontecimientos Coronarios y Otros) (40) equations for estimating CVR, which are based on the Framingham’s functions and validated in the Spanish population, will be used together with the brief neuropsychological test developed by the study NEDICES2 (41). The mediation effect of cognitive reserve and lifestyle indicators on cognitive decline in different tests of the brief neuropsychological battery of the NEDICES-2 study will also be investigated. This will make it possible to have a cohort that allows the study of the influence of cardiovascular risk on the development of cognitive impairment.

Among the strengths of this study is the type of population participating in the study. Participants will not be chosen based on their health characteristics (as in other studies where the participating population has cardiovascular risk factors or other medical characteristics), but individuals drawn from the general population will be included. In addition, the chosen age of the participants is over 54 years, while most studies have evaluated patients with a mean age of over 75 years. Including younger participants is essential to assess how the effect of cardiovascular risk factors could influence very early on the start of the neurodegenerative process that ends in cognitive impairment and dementia.

In addition, calculating cardiovascular risk is widely implemented in Spain and is performed routinely in Primary Care consultations. The demonstration that a high cardiovascular risk determines a worse cognitive performance in the Spanish population, would represent an advance in the field of dementia prevention and would allow defining the optimal target population to carry out intervention strategies, just as it is already done for prevention of cardiovascular disease, without implying the application of a new tool, since it is already used for the prevention of coronary disease.

There is little awareness that dementia risk may depend to some extent on modifiable lifestyle factors. The existence of studies in the Spanish population that support the relationship between cardiovascular risk factors and cognitive impairment could influence the approach to these vascular risk factors and the design of effective prevention strategies. In diseases such as dementia that have no treatment, prevention is a priority.

1. Prince M, Bryce R, Albanese E, Wimo A, Ribeiro W, Ferri CP. The global prevalence of dementia: A systematic review and metaanalysis. Alzheimer’s Dement [Internet]. 2013;9(1):63–75. Available from: http://dx.doi.org/10.1016/j.jalz.2012.11.007

2. World Health Organization. WHO - The top 10 causes of death [Internet]. 24 Maggio. 2018. Available from: https://public.tableau.com/views/who_ghe_mortality_top10_0/TopRankings?:incremente_view_count=no&:embed=y&:loadOrderID=0&:display_count=no&:showTabs=no&:origin=viz_share_link

3. Brookmeyer R, Gray S. Methods for projecting the incidence and prevalence of chronic diseases in ageing populations: application to Alzheimer’s disease. Stat Med [Internet]. 2000 Jun 15;19(11–12):1481–93. Available from: http://www3.interscience.wiley.com/journal/72502511/abstract%5Cnhttp://www.ncbi.nlm.nih.gov/pubmed/10844713

4. Norton S, Matthews FE, Barnes DE, Yaffe K, Brayne C. Potential for primary prevention of Alzheimer’s disease: An analysis of population-based data. Lancet Neurol. 2014;13(8):788–94.

5. Barnes D, Yaffe K. The Projected Impact of Risk Factor Reduction on Alzheimer’s Disease Prevalence. Lancet Neurol. 2013;10(9):819–28.

6. Imtiaz B, Tolppanen A-M, Kivipelto M, Soininen H. Future directions in Alzheimer’s disease from risk factors to prevention. Biochem Pharmacol [Internet]. 2014;88(4):661–70. Available from: http://linkinghub.elsevier.com/retrieve/pii/S0006295214000069

7. WHO. WHO Dementia: a public health priority. World Health Organization 2017. Available from: http://www.who.int/mental_health/neurology/dementia/en/

8. Ngandu T, Lehtisalo J, Solomon A, Levälahti E, Ahtiluoto S, Antikainen R, et al. A 2 year multidomain intervention of diet, exercise, cognitive training, and vascular risk monitoring versus control to prevent cognitive decline in at-risk elderly people (FINGER): a randomised controlled trial. Lancet (London, England) [Internet]. 2015 Jun 6;385(9984):2255–63. Available from: http://www.ncbi.nlm.nih.gov/pubmed/25771249

9. Virta, J. J., Heikkilä, K., Perola, M., Koskenvuo, M., Räihä, I., Rinne, J. O., & Kaprio, J. (2013). Midlife cardiovascular risk factors and late cognitive impairment. *European Journal of Epidemiology*, *28*(5), 405–416. https://doi.org/10.1007/s10654-013-9794-y

10. Plassman, B. L., Jr, J. W. W., Burke, J. R., Holsinger, T., & Benjamin, S. (2009). NIH Conference Annals of Internal Medicine Systematic Review : Factors Associated With Risk for and Possible Prevention of Cognitive Decline in Later Life. *Annals of Internal Medicine*.

11. Whitmer, R. A., Sidney, S., Selby, J., Johnston, S. C., & Yaffe, K. (2005). Midlife cardiovascular risk factors and risk of dementia in late life. *Neurology*, *64*(2), 277–281. https://doi.org/10.1212/01.WNL.0000149519.47454.F2

12. Gorelick, P., Scuteri, a, & Black, S. (2011). contributions to cognitive impairment and dementia a statement for healthcare professionals from the American Heart Association/American Stroke Association. *Stroke*, *42*(9), 2672–2713. https://doi.org/10.1161/STR.0b013e3182299496.Vascular

13. Livingston, G., Sommerlad, A., Orgeta, V., Costafreda, S. G., Huntley, J., Ames, D., … Mukadam, N. (2017). Dementia prevention, intervention, and care. *The Lancet*, *6736*(17). https://doi.org/10.1016/S0140-6736(17)31363-6

14. Cheng G, Huang C, Deng H, Wang H. Diabetes as a risk factor for dementia and mild cognitive impairment: a meta-analysis of longitudinal studies. Intern Med J [Internet]. 2012 May;42(5):484–91. Available from: http://www.ncbi.nlm.nih.gov/pubmed/22372522

15. Anstey KJ, Von Sanden C, Salim A, O’Kearney R. Smoking as a risk factor for dementia and cognitive decline: A meta-analysis of prospective studies. Am J Epidemiol. 2007;166(4):367–78.

16. Barreto P de S, Demougeot L, Vellas B, Rolland Y. Exercise training for preventing dementia, mild cognitive impairment, and clinically meaningful cognitive decline: a systematic review and meta-analysis. Journals Gerontol Ser A [Internet]. 2017;00(00):1–9. Available from: http://academic.oup.com/biomedgerontology/advance-article/doi/10.1093/gerona/glx234/4690262

17. Deckers, K., van Boxtel, M. P. J., Schiepers, O. J. G., de Vugt, M., Muñoz Sánchez, J. L., Anstey, K. J., … Köhler, S. (2015). Target risk factors for dementia prevention: a systematic review and Delphi consensus study on the evidence from observational studies. *International Journal of Geriatric Psychiatry*, *30*(3), 234–246. https://doi.org/10.1002/gps.4245

18. Iadecola C, Yaffe K, Biller J, Bratzke LC, Faraci FM, Gorelick PB, et al. Impact of Hypertension on Cognitive Function: A Scientific Statement From the American Heart Association. [Internet]. Vol. 68, Hypertension (Dallas, Tex. : 1979). 2016. 67–94 p. Available from: http://www.ncbi.nlm.nih.gov/pubmed/27977393%0Ahttp://www.pubmedcentral.nih.gov/articlerender.fcgi?artid=PMC5361411

19. Albanese E, Launer LJ, Egger M, Prince MJ, Giannakopoulos P, Wolters FJ, et al. Body mass index in midlife and dementia: Systematic review and meta-regression analysis of 589,649 men and women followed in longitudinal studies. Alzheimer’s Dement (Amsterdam, Netherlands) [Internet]. 2017;8:165–78. Available from: http://linkinghub.elsevier.com/retrieve/pii/S2352872917300374

20. Anstey KJ, Lipnicki DM, Low L-F. Cholesterol as a risk factor for dementia and cognitive decline: a systematic review of prospective studies with meta-analysis. Am J Geriatr Psychiatry [Internet]. 2008;16(5):343–54. Available from: http://www.ncbi.nlm.nih.gov/pubmed/18448847

21. Bermejo-Pareja, F., Llamas-Velasco, S., & Villarejo-Galende, A. (2016). Alzheimer’s disease prevention: A way forward. *Revista Clinica Espanola*, *216*(9), 495–503. https://doi.org/10.1016/j.rce.2016.05.010

22. Grau M, Elosua R, Cabrera de León A, Guembe MJ, Baena-Díez JM, Vega Alonso T, et al. [Cardiovascular risk factors in Spain in the first decade of the 21st Century, a pooled analysis with individual data from 11 population-based studies: the DARIOS study]. Rev Esp Cardiol [Internet]. 2011 Apr;64(4):295–304. Available from: https://linkinghub.elsevier.com/retrieve/pii/S0300893211001515

23. Medrano, M. J., Cerrato, E., Boix, R., & Delgado-Rodríguez, M. (2005). Factores de riesgo cardiovascular en la población española: metaanálisis de estudios transversales. *Medicina clínica*, *124*(16), 606-612.

24. Marín A, Medrano MJ, González J, Pintado H, Compaired V, Bárcena M, et al. Risk of ischaemic heart disease and acute myocardial infarction in a Spanish population: Observational prospective study in a primary-care setting. BMC Public Health. 2006;6:1–11.

25. Masiá R, Pena A, Marrugat J, Sala J, Vila J, Pavesi M, et al. High prevalence of cardiovascular risk factors in Gerona, Spain, a province with low myocardial infarction incidence. REGICOR Investigators. J Epidemiol Community Health [Internet]. 1998;52(11):707–15. Available from: http://www.pubmedcentral.nih.gov/articlerender.fcgi?artid=1756647&tool=pmcentrez&rendertype=abstract

26. Ferrieres J. The French paradox: lessons for other countries. Heart [Internet]. 2004;90(1):107–11. Available from: http://heart.bmj.com/cgi/doi/10.1136/heart.90.1.107

27. Lao O, Dupanloup I, Barbujani G, Bertranpetit J, Calafell F. The mediterranean paradox for susceptibility factors in coronary heart disease extends to genetics. Ann Hum Genet. 2008;72(1):48–56.

28. Vega T, Zurriaga O, Ramos JM, Gil M, Álamo R, Lozano JE, et al. Stroke in Spain: Epidemiologic Incidence and Patterns; A Health Sentinel Network Study. J Stroke Cerebrovasc Dis. 2009;18(1):11–6.

29. Lourida I, Soni M, Thompson-Coon J, Purandare N, Lang IA, Ukoumunne OC, et al. Mediterranean diet, cognitive function, and dementia: A systematic review. Epidemiology. 2013;24(4):479–89.

30. Harrison SL, Ding J, Tang EYH, Siervo M, Robinson L, Jagger C, et al. Cardiovascular disease risk models and longitudinal changes in cognition: A systematic review. PLoS One. 2014;9(12):1–14.

31. Dregan A, Stewart R, Gulliford MC. Cardiovascular risk factors and cognitive decline in adults aged 50 and over: a population-based cohort study. Age Ageing [Internet]. 2013 May;42(3):338–45. Available from: http://www.ncbi.nlm.nih.gov/pubmed/23179255

32. Kaffashian S, Dugravot A, Nabi H, Batty GD, Brunner E, Kivimki M, et al. Predictive utility of the Framingham general cardiovascular disease risk profile for cognitive function: Evidence from the Whitehall II study. Eur Heart J. 2011;32(18):2326–32.

33. Viticchi G, Falsetti L, Buratti L, Boria C, Luzzi S, Bartolini M, et al. Framingham risk score can predict cognitive decline progression in Alzheimer’s disease. Neurobiol Aging. 2015;36(11):2940–5.

34 DeRight J, Jorgensen RS, Cabral MJ. Composite Cardiovascular Risk Scores and Neuropsychological Functioning: A Meta-Analytic Review. Ann Behav Med. 2015;49(3):344–57.

35. Bermejo-Pareja F, Benito-León J, Vega-Q S, Díaz-Guzmán J, Rivera-Navarro J, Molina JA, et al. [The NEDICES cohort of the elderly. Methodology and main neurological findings]. Rev Neurol [Internet]. 2008;46(7):416–23. Available from: http://www.ncbi.nlm.nih.gov/pubmed/18389461

36. Hernández-Gallego J, Llamas-Velasco S, Bermejo-Pareja F, Vega S, Tapias-Merino E, Rodríguez-Sánchez E, et al. Neurological Disorders in Central Spain, Second Survey: Feasibility Pilot Observational Study. JMIR Res Protoc 2019;8(1)e10941 https//www.researchprotocols.org/2019/1/e10941/ [Internet]. 2019 Jan 10 [cited 2019 Feb 8];8(1):e10941. Available from: https://www.researchprotocols.org/2019/1/e10941/

37. Llamas-Velasco S, Contador I, Villarejo-Galende A, Lora-Pablos D, Bermejo-Pareja F. Physical Activity as Protective Factor against Dementia: A Prospective Population-Based Study (NEDICES). J Int Neuropsychol Soc [Internet]. 2015 Nov 19 [cited 2018 Feb 14];21(10):861–7. Available from: http://www.journals.cambridge.org/abstract_S1355617715000831

38. Contador I, Bermejo-Pareja F, Puertas-Martin V, Benito-Leon J. Childhood and Adulthood Rural Residence Increases the Risk of Dementia: NEDICES Study. Curr Alzheimer Res [Internet]. 2015 Apr 27;12(4):350–7. Available from: http://www.eurekaselect.com/openurl/content.php?genre=article&issn=1567-2050&volume=12&issue=4&spage=350

39. Marrugat J, Vila J, Baena-Díez JM, Grau M, Sala J, Ramos R, et al. [Relative validity of the 10-year cardiovascular risk estimate in a population cohort of the REGICOR study]. Rev Esp Cardiol [Internet]. 2011;64(5):385–94. Available from: http://www.revespcardiol.org/es/validez-relativa-estimacion-del-riesgo/articulo/90003647/

40. Marrugat, J., Subirana, I., Ramos, R., Vila, J., Marín-Ibañez, A., Guembe, M. J., … Elosua, R. (2014). Derivation and validation of a set of 10-year cardiovascular risk predictive functions in Spain: The FRESCO Study. *Preventive Medicine*, *61*, 66–74. https://doi.org/10.1016/j.ypmed.2013.12.031

41. Serna, A., Contador, I., Bermejo-Pareja, F., Mitchell, AJ, Fernandez-Calvo, B., Ramos, F., ... & Benito-Leon, J. (2015). Precisión de una batería neuropsicológica breve para el diagnóstico de demencia y deterioro cognitivo leve: un análisis de la cohorte NEDICES. *Diario de la enfermedad de Alzheimer* , *48* (1), 163-173.

**Hypothesis**

Higher cardiovascular risk in the population between 55 and 74 years old, measured with the Framingham REGICOR and FRESCO risk equations, is related to a worse score at 5 years of follow-up in the Brief Neuropsychological Battery used in the NEDICES2 study.

**Objectives**

**Primary aim**

To determine the relationship between CVR, measured with the Framingham REGICOR and FRESCO risk equiations, and the change in cognitive performance after 5 years in the subjects from 55 to 74 years of age included in NEDICES2-RISK study.

**Secondary aims**

1. To describe the profile of patients based on their CVR.
2. To assess the association between each of the studied CVR factors and the change in cognitive performance after 5 years.
3. To analyze potential mediating factors between CVR, cognitive performance and lifestyle.
4. To assess the effect of indicators of CR (verbal intelligence, education, occupation, and lifestyle) on cognitive performance, as measured via different neuropsychological tests.

**Methods/design**

**Design**. Observational, analytic, prospective cohort study with a 5-year follow-up.

**Ambit**. Patient recruitment took place in the primary care setting within the Spanish National Health System in the regions of Ávila, Madrid, Salamanca, and Segovia, Spain.

**Population**. The studied population are patients 55 to 74 years old, already included in the cohort of the former study NEDICES2 (2014-2017). The population in the study NEDICES2 originated from the list of users (social security card holders) of the participating doctors in the included healthcare centers, who were then selected following randomized sampling of patients ≥55 years-old stratified by gender and age (5-year intervals).

**Selection criteria**

1. Criteria for inclusion

- Age from 55 to 74 years
- Providing informed written consent for their inclusion in the study

1. Criteria for exclusion

- Presenting a diagnosis of dementia at baseline

**Sample size**: 1955. All subjects in the NEDICES2 study

**Variables**

1. Socio-demographic variables: age, gender, educational level, current occupation, marital status, weight at birth, family unit, and family background. Health habits: sleep hours, alcohol consumption, tobacco consumption, physical activity and Mediterranean diet. Depression questionnaire: CES-D. Current chronic diseases and treatments collected and confirmed by family doctors. Word stress test.
2. Exposure variables: Risk cardiovascular factors. Cardiovascular risk measured with the Framingham REGICOR and FRESCO risk equiations at the time of the first neuropsychological assessment carried out in NEDICES2 (2014) and 5 years later.
3. Main outcome variables: 37-item version of the Mini-Mental State Examination (MMSE-37), immediate and differed memory measured via the SEN-FIS illustrations, 11-item version of Pfeffer’s Functional Activities Questionnaire (FAQ), Katz index of Independence in Activities of Daily Living (ADL), Trail Making Test, oral fluency test by categories (category: animals), word stress test, and clock-drawing test.

**Data collect**

Data collection is performed at two different times:

1. Variables recorded in the NEDICES2 study 3 years ago (2014):
   1. Interviews and questionnaires carried out by the family doctor in the NEDICES2 study: chronic diseases and medication prescribed in the last month
   2. Trained interviewers: sociodemographic variables and neuropsychological tests
   3. Exposure variable: the calculation of cardiovascular risk will be made with the above data
2. Variables collected in 2019:
   1. Cardiovascular risk factors collected by family doctors in 2019
   2. Trained interviewers: sociodemographic variables and neuropsychological tests
   3. Exposure variable: new calculation of cardiovascular risk in 2019

**Statistical analysis**

1. The quality of data will be checked: codification errors, missing data, and potential biases related to the representativeness of the sample.
2. Descriptive analysis. Socio-demographic and clinical variables of subjects: frequencies and percentages in the cases of categorical variables, and by their means (standard deviation) or median (interquartile range) in the case of quantitative variables.
3. Bivariate analysis: relationship between basal CVR (four categories: low, medium, high and very high cardiovascular risk) and the score obtained in the neuropsychological tests, MMSE-37, immediate and differed memory measured via the SEN-FIS illustrations, 11-item version of Pfeffer’s Functional Activities Questionnaire (FAQ), Trail Making Test, oral fluency test by categories (category: animals) and clock-drawing test.
4. Multivariate analysis: relationship between basal CVR and change in neuropsychological tests adjusted by eelevant sociodemographic and clinical variables and potential confounding factors using ANCOVA. linear regression multilevel mixed-effects models will be adjusted to account for intra-subject variability (by taking repeated measures) and clustering of patients.
5. The reliable change will be analyzed for each test in independent samples (high cardiovascular risk vs low cardiovascular risk) using the method by Hsu and Chelene. Normalized change tables will be presented for each of the groups controlling the effect of sociodemographic variables.

**Limitations**

Information from the clinical file of patients will be used in the cases of missing data. Completing data from this secondary source of information can affect the quality of data of variables like blood pressure, weight, height, or lifestyle, since they may be also missing or because of the potentially increased variability resulting from multiple professionals collecting them. However, working with electronic clinical files, a system that has been implemented for over 15 years, guarantees the quality and homogeneity of the data they contain.

CVR factors can change throughout the studied period, due to no longer being exposed to them or the appearance of new ones. To avoid the error these changes can produce, CVR will be estimated at two time points, namely at the first neuropsychological evaluation and five years later at the second one, and the possible variations between them will be examined.

**Ethical considerations and data confidentiality**

The informed consent of the patients for the NEDICES2 study is available, permission will be requested to review the documentation and clinical history of the participants from the Research Ethics Committee of the 12 de Octubre Hospital.

There is a web page aimed at patients with the aim of providing them with information related to the study, as well as providing the main findings and results of the study.

The estimations of CVR and MMSE-37 will be handed over to the primary healthcare physicians responsible for the included patients in order to adjust their treatment if necessary or further their evaluation of cognitive performance.

A local database will be created to record all the obtained data. Upon finalization of this study, this database will be sent to the central database of the NEDICES2 study, and one copy will be kept at the Department of Neurology of Hospital 12 de Octubre. The nominative database will be protected by passwords only available to the researchers.

The confidentiality and anonymity of the data will be ensured according to Law 15/1999 on data confidentiality, both in the execution phase of the project and in the presentations or publications derived from it.
